# Mitochondrial DNA haplogroup H structure in North Africa

**DOI:** 10.1186/1471-2156-10-8

**Published:** 2009-02-25

**Authors:** Hajer Ennafaa, Vicente M Cabrera, Khaled K Abu-Amero, Ana M González, Mohamed B Amor, Rym Bouhaha, Nduna Dzimiri, Amel B Elgaaïed, José M Larruga

**Affiliations:** 1 Laboratory of Genetics, Immunology and Human Pathology at the Faculty of Sciences of Tunis, Faculty of Sciences of Tunis, University El Manar I, 1060 Tunis, Tunisia; 2 Department of Genetics, Faculty of Biology, University of La Laguna, Tenerife 38271, Spain; 3 Molecular Genetics Laboratory, College of Medicine, King Saud University, Riyadh, Saudi Arabia; 4 Department of Genetics, King Faisal Specialist Hospital, Riyadh, Saudi Arabia

## Abstract

**Background:**

The Strait of Gibraltar separating the Iberian Peninsula from North Africa is thought to be a stronger barrier to gene flow for male than for female lineages. However, the recent subdivision of the haplogroup H at mitochondrial DNA (mtDNA) level has revealed greater genetic differentiation among geographic regions than previously detected. The dissection of the mtDNA haplogroup H in North Africa, and its comparison with the Iberian Peninsula and Near-East profiles would help clarify the relative affinities among these regions.

**Results:**

Like the Iberian Peninsula, the dominant mtDNA haplogroup H subgroups in North Africa are H1 (42%) and H3 (13%). The similarity between these regions is stronger in the North-West edge affecting mainly Moroccan Arabs, West Saharans and Mauritanians, and decreases eastwards probably due to gene flow from Near East as attested for the higher frequencies of H4, H5, H7, H8 and H11 subgroups. Moroccan Berbers show stronger affinities with Tunisian and Tunisian Berbers than with Moroccan Arabs. Coalescence ages for H1 (11 ± 2 ky) and H3 (11 ± 4 ky) in North Africa point to the possibility of a late Palaeolithic settlement for these lineages similar to those found for other mtDNA haplogroups. Total and partial mtDNA genomic sequencing unveiled stronger mtDNA differentiation among regions than previously found using HVSI mtDNA based analysis.

**Conclusion:**

The subdivision of the mtDNA haplogroup H in North Africa has confirmed that the genetic differentiation found among Western and Eastern populations is mainly due to geographical rather than cultural barriers. It also shows that the historical Arabian role on the region had more a cultural than a demic effect. Whole mtDNA sequencing of identical H haplotypes based on HVSI and RFLP information has unveiled additional mtDNA differences between North African and Iberian Peninsula lineages, pointing to an older mtDNA genetic flow between regions than previously thought. Based on this new information, it seems that the Strait of Gibraltar barrier affected both male and female gene flow in a similar fashion.

## Background

Bounded by the Mediterranean Sea in the North and the Sahara desert in the South, North Africa behaved as an anthropological island in the African Continent. Previously, only the Strait of Gibraltar in the West and the Suez Isthmus in the East connected this region to Europe and the Near East respectively. These two passageways were the most probable migratory routes followed by human intercontinental dispersals since prehistoric times. In addition, periodic wetter climatic conditions allowed contacts with sub-Saharan African peoples across the Sahara desert and seafaring achievement brought numerous Mediterranean and Atlantic cultures to the North African shores [1]. Archaeological information points to a modern human occupation of this area since 45,000 years ago (ya), as attested by the Aterian industry [2]. However, there is no unanimous assent about the degree of human continuity since that time, as some of the posterior Palaeolithic industries (Iberomarusian, Dabban) exhibit no clear cultural connections with the earlier Aterian form. Furthermore, there is also controversy about the demic impact of the Near East Neolithic on the Northwest African autochthonous Capsian Neolithic [1]. Even more tenuous are the putative connections of the Aterian with the Solutrean of Iberia, or those of the Capsian with the Mediterranean Neolithic [3]. Nonetheless, it is agreed that the historic penetration in the area of the Pharaonic and classical Mediterranean cultures, ending with the Islamic domination, imposed strong cultural influences with only a minor demic impact [4,5]. Population genetic studies using classical markers pointed to a sizeable Upper Paleolithic component in Northwest African populations [6], whereas the Neolithic diffusion in that region was more a cultural than a demic process [7]. More recently, the haploid characteristics of the uniparental genetic markers allowed the successful application of phylogenetic and phylogeographic approaches to population genetics. Thus, mitochondrial DNA (mtDNA) phylogeographic analyses have enhanced the power of this maternal non-recombining marker to detect human migrations on continental [8-10] and regional [11-14] scales. Focusing on North Africa, several mtDNA studies have shown that, in spite of an important Sub-Saharan African contribution, the majority of the lineages detected in this region belong to, or have common roots with, Eurasian haplogroups [15-23]. Some of these haplogroups, including the X1 [12], U6 [11,13] and M1 [13,14], although of West Asian origin, have Paleolithic coalescence ages in North Africa. Others seem to be of more recent acquisition as a result of European (U5, V [24-26]) or Middle Eastern influences (R0a, J1b, U3 [17,27-29]). In agreement with classical markers and mtDNA, in an early analysis of Northwest African populations using paternal Y-chromosome variation, it was proposed that the main haplogroups defined by the M78 and M81 binary markers could be the paternal counterparts of the classical and maternal Paleolithic components [30]. However, more recent studies in which those and other markers were further subdivided suggested a predominantly Neolithic origin for the Y-chromosomal DNA variation in North Africa [20,22-34].

The discrepancies in the uniparental marker results could be due to real differences in male and female demographic histories [35]. However, a lack of mtDNA haplogroup resolution could also be responsible. Recently, the accurate dissection of the most frequent Western Eurasian haplogroup H into several monophyletic subhaplogroups [36,10][37-40] changed a rather uniform genetic landscape into one with several regional peaks and clinal variations. Thus, the frequencies of H1 and H3 subhaplogroups are highest in Western Europe, decreasing gradually to the East. In contrast, H2 occurs more frequently in Eastern than in Western Europe. Besides, there are subhaplogroups that characterize other regions, such as H6 and H8 in Central Asia, H13 in the Near East, H20 and H21 in the Caucasus, and H18 in the Arabian Peninsula. Haplogroup H is also the most frequent clade in North Africa. Global frequencies are highest in the Northwest, representing 37% and 34% of their mtDNA lineages in Berber and Arab speaking Moroccans [15], 24% [41] and 32% [19] in Berber Algerian samples and 26% in Tunisian Berbers [20]. The frequencies drop slightly southwards, showing 24% in Saharans [15] and 23% in Mauritanians [42], as well as eastwards displaying 21% [16] and 14% [21] in Egyptian samples. The aims of this paper are: 1) to subdivide the North African haplogroup H lineages into its known subhaplogroups, 2) to establish the phylogenetic and phylogeographic patterns of these subhaplogroups in the region, and 3) to compare them with those present in Europe and the Near East, in order to establish the strength of the human migrations from both continents into North Africa in spatial and temporal dimensions.

## Results

**Figure 1:**
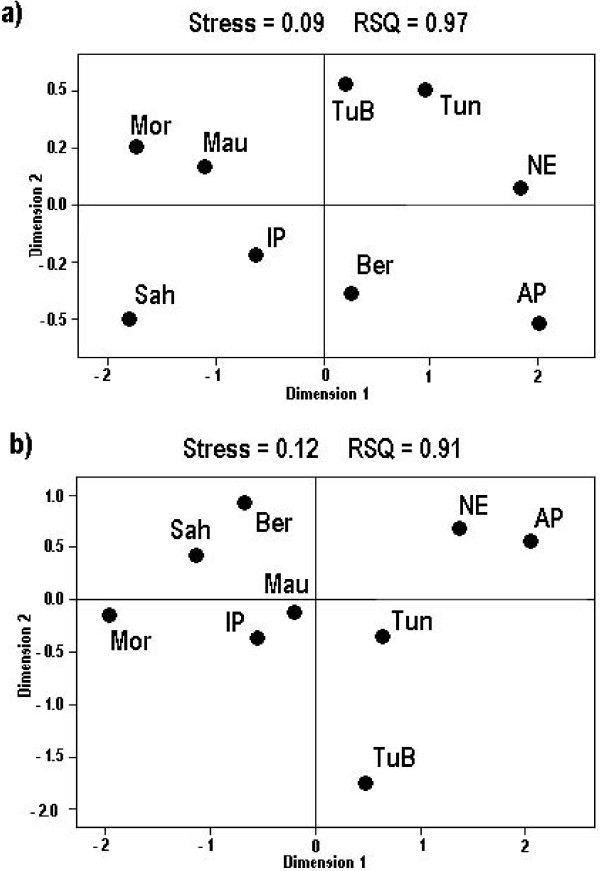
**Graphical relationships among the studied populations**. Codes are as in Table 1. MDS plots based on F_ST _haplogroup (a) and haplotypic (b) frequency distances.

As described previously, total frequencies for the haplogroup H decline toward both the East and the South (Table 1). The haplogroup H represents 44% of the mtDNA variation in the Iberian Peninsula, but only 22% in the Near East. Likewise, this distribution still reaches 25% in North Africa, but drops to only 9% in the Arabian Peninsula. Haplogroup H subclade distribution is also very different in the various regions. Subhaplogroups H1 and H3 are the dominant subgroups in the Iberian Peninsula (45% and 16%, respectively) and North Africa (42% and 13%, respectively) whereas unclassified H haplotypes (H*) account for 40–50% of the H diversity in the Arabian Peninsula and the Near East. Furthermore, while H1 (12%) is still the most frequent subgroup, followed by the H5 (8%) in the Near East, the modal subclades in the Arabian Peninsula are H2a1a (18%) and H6b (14%). Pairwise F_ST _distances based on sub-haplogroup frequencies display a high heterogeneity among the main regions (Table 2). However, the level of statistical significance between the Iberian Peninsula and North Africa (p < 0.05) is lower than that for any other pairwise comparison (p < 0.001). In addition, within North African populations, the Tunisians, Tunisian Berbers and Moroccan Berbers are different from the Saharan and Moroccan Arabs, while the last two are comparatively less different from the Iberian Peninsula. The relative proximity of the Iberian Peninsula to the westernmost North African populations is graphically reflected in Figure 1a. It is evident that Tunisians and Berbers are closest to the Near East and the Arabian Peninsula. A principal component analysis (PCA) points to subhaplogroups H1 and H3 as being primarily responsible for the Iberian-Moroccan-Saharan connection, whereas H4, H5, H7, H8 and H11 testify the Near East influence (data not shown). Similarly, haplotypic based F_ST _distances show a strong influence of the Iberian Peninsula on the Western Moroccan and Saharan North African populations, and indicate that Tunisians are comparatively the most remarkably influenced by the Near East (Table 2 and Figure 1b). Globally, North Africa shares a similar number of haplotypes with the Iberian Peninsula compared with the Near East (Table 3). However, a detailed analysis of the ratios between haplotypic identities relating each North African population with the Iberian Peninsula or the Near East confirms that the Western populations, comprising Moroccan Arabs, Saharans and Mauritanians, are the most notably influenced by the Iberian Peninsula, whereas the Tunisian Berbers, Tunisians, and the Moroccan Berbers have received relatively more gene flow from the Near East (Table 3). At this point, it is noteworthy that all the Arabian Peninsula haplotypes shared with North Africa are a subset of those shared by the latter with the Near East, pointing to a minor direct input of the Arabian Peninsula on the North African populations. Haplogroup (Table 1) and haplotype (Table 3) genetic diversities demonstrate that the Northwestern African populations (Moroccan Arabs and Saharans) are genetically less diverse than the more central Tunisian and Berbers, a fact that could be explained by a stronger Near East influence on the later populations. Although global haplogroup and haplotypic diversities are not statistically different among regions (Table 1 and 3), the European subgroup H1 appears to be significantly more diverse in the Near East (87 ± 5) than in the Iberian Peninsula (75 ± 3) or North Africa (67 ± 6). Moreover, the genetic diversity for the Western European subgroup H3, which is absent in the Near East, is also higher in North Africa (74 ± 9) than in the Iberian Peninsula (65 ± 6). Transformation of molecular genetic diversities in coalescence ages gives 18,345 ± 4,051, 14,201 ± 2,984, and 11,366 ± 2,354 years for H1 in the Near East, Iberian Peninsula and North Africa, respectively. On the other hand, the coalescence ages for H3 in the Iberian Peninsula (10,342 ± 2,634) and North Africa (10,866 ± 4,107) are similar. However, only H1 ages in Near East and North Africa are statistically different from each other.

**Table 1:** Distribution of subhaplogroup H frequencies (%) in the studied populations.

	**Populations**^1^									
**Subhaplogroup**	**IP**	**Mau**	**Sah**	**Mor**	**MoB**	**TuB**	**Tun**	**NA**	**AP**	**NE**
H*	18	18	14	11	30	21	48	26	43	51
H1	45	64	68	63	30	24	24	42	6	12
H1a	1	-	-	-	-	-	-	-	-	1
H1b	2	-	-	-	4	-	-	1	1	1
H2a	4	-	-	-	4	-	-	1	2	1
H2a1a	1	-	-	-	-	-	-	-	18	1
H3	16	9	7	17	4	21	15	13	-	-
H4	3	-	-	-	17	7	2	5	4	4
H5	1	-	-	3	2	3	4	2	3	8
H5a	5	-	-	3	-	-	-	< 1	1	2
H6	< 1	-	-	-	-	-	-	-	-	1
H6a	3	-	4	-	4	-	-	1	3	1
H6b	-	-	7	-	-	-	-	1	14	2
H7	1	9	-	-	2	17	7	5	3	4
H8	-	-	-	-	-	3	-	< 1	-	1
H11	< 1	-	-	3	-	3	-	1	-	1
H13a1	1	-	-	-	-	-	-	-	1	4
H14a	-	-	-	-	-	-	-	-	1	4
H20	< 1	-	-	-	-	-	-	-	1	2
										
H classified	**510**	**11**	**28**	**35**	**46**	**29**	**46**	**203**	**148**	**253**
N_H_^2^	593	18	30	42	49	29	46	224	149	265
N^2^	1349	102	128	180	154	101	186	880	1685	1201
%H	**44**	**18**	**23**	**23**	**32**	**29**	**25**	**25**	**9**	**22**
h_HG _± se^3^	74 ± 2	60 ± 15	53 ± 10	58 ± 9	79 ± 3	85 ± 3	70 ± 5	74 ± 2	76 ± 3	72 ± 3

1. IP = Iberian Peninsula; Mau = Mauritania; Sah = Sahara; Mor = Morocco; MoB = Moroccan Berbers; TuB = Tunisian Berbers; Tun = Tunisia; NA = North Africa; AP = Arabian Peninsula; NE = Near East.

2. Haplogroup H (N_H_) and total (N) sample sizes.

3. Gene haplogroup diversity and standard error (h_HG _± se), in percentage.

4. < 1 = subhaplogroup present in frequency of less than 1%.

**Table 2:** F_ST _(by 1,000) based on subhaplogroup, above the diagonal, and haplotype, below the diagonal, frequencies.

	Tun	TuB	MoB	Mor	Sah	Mau	**NA**	**IP**	**AP**	**NE**
Tun	-	33	30*	200***	227***	129**		86***	61***	20*
TuB	7	-	22	109***	152***	56		36***	11***	100***
MoB	17*	48***	-	115***	121**	55		38***	83***	61***
Mor	40***	51***	16*	-	0	0		14	334***	308***
Sah	24*	45**	5	0	-	0		31*	334***	316***
Mau	0	11	0	0	0	-		0	260***	231***

**NA**								6*	145***	116***

**IP**	10**	24***	13***	13*	4	0	3*	-	192***	170***
**AP**	18***	49***	47***	80***	55***	29*	42***	42***	-	34***
**NE**	6	41***	28***	64***	40***	14	28***	30***	13***	-

*= p < 0.05, ** = p < 0.01, *** = p < 0.001

**Table 3:** Population and regional haplotypic composition.

	**HT**	**HT**_U_	**%h**_HT_	**%HT**_U_	**%sHT**_NA_	**%sHT**_IP_	**%sHT**_TNE_	**%sHT**_C_	**IP I**_HT_	**TNE I**_HT_	**IP/TNE**
**IP**	161	122	93 ± 1	76							

Mau	9	1	95 ± 7	11	22	22	-	44	0.414	0.238	1.74
Sah	16	6	89 ± 5	38	-	31	6	25	0.349	0.167	2.09
Mor	20	9	86 ± 6	45	15	20	5	15	0.217	0.107	2.03
MoB	20	5	90 ± 3	25	15	-	15	45	0.280	0.321	0.87
TuB	15	4	93 ± 3	27	-	20	13	40	0.373	0.285	1.31
Tun	30	12	97 ± 1	40	7	13	17	23	0.228	0.214	1.06

**NA**	80	43	93 ± 1	54		16	13	18	0.210	0.160	1.31
**AP**	58	36	96 ± 1	62							
**NE**	129	90	97 ± 1	70							

Number of different haplotypes (HT), unique haplotypes (HT_U_), haplotype diversity (± standard error) (%h_HT_), unique haplotype frequency (%HT_U_). Frequency of North African haplotypes shared only with other North Africans (%sHT_NA_), IP (%sHT_IP_), NE and/or AP (%sHT_TNE_), and with both IP and TNE regions (%sHT_C_). North African haplotypic identity, by 100, with IP (IP I_HT_) and with TNE (TNE I_HT_) and ratio between them (IP/TNE).

**Figure 2:**
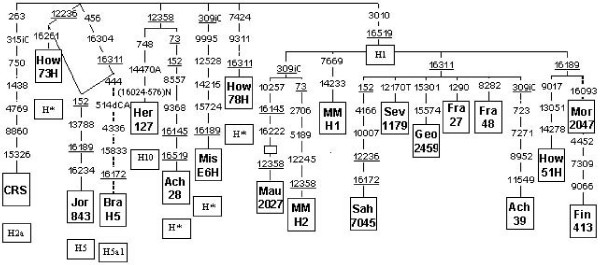
**Phylogenetic tree of complete (continuous branches) or nearly complete (discontinuous branches) haplogroup H mtDNA sequences**. Numbers along links refer to nucleotide transitions. "A" and "T" indicate transversions; "d" deletions and "i" insertions. Recurrent mutations are underlined. The empty box represents a node from which other (not shown) sequences branch. Sequence references are: CRS [64,65]; How 73H, How 78H and How51H [66]; Bra H5 [49]; Her 127 ([10], EF657262); Ach 28 and Ach 39 ([37], AY738967 and AY738978); Mis E6H [62], AY195757); MM H2 and MM H1 ([9], AF382002 and AF381993); Fra 27 and Fra 48 ([67], DQ523627 and GQ523648); Fin 413 ([36], AY339413); Jor 843, Mau 2027, Sah 7045, Sev 1179, Geo 2459, Mor 2047 (present study). Geographic origins are: How 73H, How 78H and How51H: Dutch, Jor 843: Jordan, Bra H5 and Her 127: Europeans, Ach 28 and Ach 39: Italians, Mis E6H: Israeli, Mau 2027 and MM H1: Mauritanians, MM H2 and Sev 1179: Spaniards, Sah 7045: West Saharan, Geo 2459: Georgian, Fra27 and Fra 48: Sardinians, Mor 2047: Moroccan, Fin 413: Finlander.

The relative affinities among regions are based on subhaplogroup frequencies, which do not take into account differences between haplotypes assorted in the same subgroup, or in haplotypic matches, whose identity is based only on partial HVSI sequences. In addition, it has to be taken into account that half of the H lineages detected in North Africa are not shared with other regions and that this percentage is even greater in the putative source regions of the Near East (70%) and the Iberian Peninsula (76%). These facts point to a higher differentiation among regions and between populations than those observed previously. Indeed, complete or nearly complete sequencing of some apparently identical samples indicates that the real genetic heterogeneity among regions is greater than those estimated above (Figure 2). To begin with, the HVSI motif 16093 -16189 that characterizes subgroup H1f was found in an individual (Mor 2047) from Morocco (Figure 2) also in an H1 background. This sub-group is particularly abundant and mainly restricted to Finland and the surrounding populations [36]. At first sight, this coincidence would seem to point to a new link between North European with North African populations like that found previously for U5b1b [26]. However, in this case, further analysis of the coding region in the North African sample revealed a lack of the three coding region mutations that additionally characterize the Finish H1f subgroup [38] (Figure 2). This lack of identity between haplotypes assorted in the same subgroup and sharing the same or similar HVSI motif can be extended to other cases. For instance, there is a group of H sequences that shares the 16145 – 16222 HVSI motif consistently found in Northwestern Africa, the Sahara and several Western Sahelian populations [15]. The complete sequencing of a Mauritanian sample (Mau 2027) allowed the assignation of this type to the subhaplogroup H1 (Figure 2). The direct connection of this motif with a German sequence was previously suggested [15]. However, the additional presence of transitions 16304 and 456 in the HVSI and HVSII regions respectively in that German haplotype [43] indicated that it should be classified as belonging to the H5 instead of the H1 subgroup, which does not support a direct link between these regions. In contrast, the two 16145 – 16222 haplotypes sporadically detected in the Iberian Peninsula [[44] and unpublished results] belonged to the North African subgroup as they shared the coding 10257 mutation, in addition to the H1 diagnostic transition 3010, with the totally sequenced Mauritanian sample (Figure 2). It seems that the 10257 transition defines a new subgroup within H1. This fact points to a possible, although not recent, North African demic influence on the Iberian genetic pool. Another interesting group of sequences belonging to the H1 subgroup in North Africa is that characterized by the 16172 – 16311 motif, which we [15] and others [19] have found mainly in Saharan samples. Haplotypes with, or including, this HVSI motif have also been detected in European [45,43,8][46-49] and in Asian [50-54] samples, but not in the Iberian Peninsula yet (see Additional file 1). However, the possibility of direct phylogenetic links among such distant regions is very weak, because all of those individuals further classified in both regions belong to the H5 subgroup or the HV haplogroup [48,49] in Europe, or to the HV or the R2 haplogroups [53,54] in the Middle East, which strongly points to yet another case of HVSI convergence in distinct backgrounds of coding regions. In addition to the CRS, the 16189 and the 16311 HVSI motifs are quite abundant in North Africa (see Additional file 1). However, when these samples were screened for the coding region positions observed in completely sequenced European or Middle East individuals that held the same HVSI motifs (Figure 2), none of these positions appeared in the North African samples. This lack of homogeneity again strongly points to their different monophyletic coding backgrounds, in spite of their HVSI matches, a fact repeatedly found in other studies [38]. Indeed, in this study, there are also instances of molecular convergence in the coding region. Sequences How 73H and Jor 843 share the 12236 transition, although they respectively belong to the H* and H5 subgropups (Figure 2). The 12358 transition also presents one such case that is shared by four sequences (Her 127, Ach 28, MM H2, and Mau 2027) belonging to different H subgroups (Figure 2).

## Discussion

The dissection of mtDNA haplogroup H in North Africa has confirmed several genetic features of its populations. First, there is a significant genetic differentiation between Northwestern, Central and Eastern populations already detected since the first genetic studies carried out in North Africa using classical genetic polymorphisms [6]. This differentiation has also been found by posterior molecular analyses using Y-chromosome markers [31-34] or X-chromosome SNPs [55]. Second, as Arab and Berber communities are present in both areas, geographic isolation, more than cultural barriers, seems to be the main cause of this genetic differentiation. This has been consistently reported in all previous studies using autosomal short tandem repeats [4], autosomal Alu insertion polymorphisms [56,57], high-resolution Y-chromosome analyses [30,58,59], and mtDNA polymorphisms [19,20,22,23]. As a consequence, it has been proposed that the North African gene pool has had Palaeolithic and Neolithic influences from the East, but that the impact of the historical invasions, such as the Arabic role, had more a cultural than a demic effect. The lack of exclusive haplotypic matches between North Africa and the Arabian Peninsula found here is in accordance with that hypothesis. Third, the southward clinal diminution of haplogroup H frequencies found at mitochondrial level is well explained as a counteracting effect of the northward clinal diminution of the Sub-Saharan maternal gene flow [15,5,19]. Fourth, the genetic heterogeneity detected between the North African and the Iberian Peninsula populations has been attributed to both the effect of the physical barrier imposed by the Strait of Gibraltar and strong cultural differences. However, some gene flow has been detected between areas and its strength depends mainly on the type of marker used. The strongest barrier effect has been detected in analyses based on Y-chromosome polymorphisms [30]. The levels of gene flow detected in autosomal studies have been of more diverse range [4,56] and, in some cases, seem to depend on the population samples used as is the case with, for instance, the CD4/Alu microsatellite haplotypes [60,61]. In contrast, a high female permeability has been deduced from several mitochondrial studies that pointed to the existence of an important maternal Iberian input on North Africa [15,19]. Although there is no archaeological evidence to justify such a demic flow from Iberia to North Africa, based on the phylogeographic range, comparative gene diversity and ages of several mitochondrial haplogroups such as V, H1, H3, and U5b1b [25,37,26], the presence of these haplogroups in North Africa is thought to be the result of a southward expansion of Palaeolithic hunter-gatherers from the Franco-Cantabrian refuge after the Last Glacial Maximum. In fact, coalescence ages for H1 and H3 subclades estimated in this study are in good agreement with those previously published and are congruent with these expansions. Thus, our HVSI based coalescence ages for H1 (14.2 ± 3.0 ky) and H3 (10.3 ± 2.6 ky), in the Iberian Peninsula, are very close to those published by Pereira et al. [40] in the same area for H1 (14.0 ± 3.0 ky) and for all of Europe for H3 (11.0 ± 3.0 ky). Furthermore, striking similarities are observed when these ages are compared to those obtained from the coding region in similar geographic ranges, using the Mishmar et al. calibration [62]. Thus, H1 coalescence ages for Iberia (13.0 ± 6.0 ky; [40]) and Southwest Europe (12.8 ± 2.4 ky; [37]) are very similar between themselves, and not significantly different from those based on the HVSI. Likewise, H3 coding region based coalescence ages for whole Europe (9.0 ± 3.0 ky; [40]) and Southwest Europe (10.3 ± 2.4 ky; [37]) are also very similar to those based only on the HVSI. That Palaeolithic expansion would explain the notorious presence of H1 and H3 detected mainly in the most North-western populations of North Africa and the decrease in their frequency eastwards. However, if this hypothesis held, the comparatively high diversity of H1 and H3 in North Africa would point to an important Palaeolithic gene flow from the Iberian Peninsula to North Africa across the Strait of Gibraltar. On the contrary, a consensus exists regarding the Near East origin of the bulk of the Y-chromosome and mtDNA North African lineages. However, discrepancies still exists with respect to the time in which these settlements most probably occurred. In the first Y-chromosome pioneering studies of the region, a Palaeolithic settlement for the autochthonous E-M81 clade was hypothesized in accordance with the age proposed based on classical markers [30]. However, later studies have assigned this, and other subclades derived from E-M78, that are particularly abundant in North Africa, a Neolithic or even historic settlement age and a Near East or Northeast African source [63,31-34]. On the other hand, for those mtDNA haplogroups pre-eminent in North Africa, that have been analyzed at deep genomic and phylogeographic levels, such as U6 and M1, a Palaeolithic settlement and Middle East roots have been proposed [11,13,14]. From our data, it can be also deduced that the presence of the H1 and H3 subgroups in North Africa could have similar expansion times as in Europe and, therefore, a late Palaeolithic settlement in the region. Finally, it should be noted that the different levels of gene flow detected throughout the Strait of Gibraltar, with respect to Y-chromosome and mtDNA polymorphisms have been attributed to sexual migratory differences, with females showing more permeability than males due to patrilocality and polygyny [5,60,19], and to genetic drift differently affecting both sexes [22,59]. However, the first explanation is not in accordance with the demographic flows known to have occurred between Morocco and Iberia across the Strait of Gibraltar. Historically, the main human movement from Northwest Africa to the Iberian Peninsula was the Islamic Invasion. As a military enterprise, it is believed that this North African gene flow into Iberia was mainly a male contribution. If genetically important, it would homogenize the male lineages between Iberia and North Africa to a greater extent than the female lineages, in contradiction to the experimental results. Little is known about prehistoric contacts between these two areas, but human movements repeatedly crossing the Gibraltar Strait to establish patrilocality seems improbable. The lack of deep sequence identity for several mtDNA haplotypes assorted in the same H subgroup and considered haplotypic matches between North Africa and the Iberian Peninsula, clearly points to the existence of a higher mtDNA heterogeneity between these two regions than suggested in previous studies. If the greater level of differentiation established for H in the present study were extendable to other mitochondrial haplogroups, the female levels of gene flow between both areas would match approximately those of males. Further mtDNA studies at genomic level are necessary to test this hypothesis.

## Conclusion

The subdivision of mtDNA haplogroup H in North Africa has confirmed that the genetic differentiation found among Western and Eastern populations is mainly due to geographical rather than cultural barriers. It also appears that the historical Arabian role on the region had more a cultural than a demic effect. Whole mtDNA sequencing of apparently identical H haplotypes, based on HVSI and RFLP information, has unveiled additional mtDNA differences between North Africa and the Iberian Peninsula, pointing to the Strait of Gibraltar barrier as affecting male and female gene flow in a similar fashion.

## Methods

A total of 5,115 mtDNA sequences were analyzed. Of these, 1,231 belonged to the haplogroup H, defined as -7025 AluI by RFLP screening. Four main geographic areas were covered by this study: the Iberian Peninsula (593 H individuals from a total of 1,349), North Africa (224 H individuals from a total of 880), the Near East (265 H individuals from a total of 1,201) and the Arabian Peninsula (149 H individuals from a total of 1,685). Detailed origin and geographic localization for all the samples are specified in Additional file 2. From the 1,231 individuals classified as H, 1,114 could be assorted into one of 19 different H subgroups by further screening for characteristic HVSI and/or HVSII sequence motifs, or diagnostic RFLPs (see Additional file 3). Analyzed individuals that could not been assorted into any of the known groups were considered as H* types. In addition, complete or nearly complete mtDNA sequencing was carried out on 6 individuals with haplotypes found only in well-defined geographic areas or with HVSI haplotypic matches between very distant regions with the aim of accessing whether these matches also held for their coding regions (Figure 2, [64,36,9,10,62,37,67,49]). Furthermore, in order to find out additional subdivisions, those individuals presenting HVSI matches with already published complete haplogroup H sequences were screened for all coding region positions they hold (Figure 2). DNA extraction, primers, conditions used for PCR amplifications and total or partial sequencing have been published previously [9,29]. RFLP analyses and subhaplogroup H nomenclature (see Additional file 3) were as in Loogväli et al. [38] and Roostalu et al. [68]. Haplogroup and haplotype diversities (h) as well as molecular genetic diversities (π) were calculated according to Nei et al. [69]. Only HVSI positions from 16,024 to 16,365 were used for genetic comparisons of partial sequences with other published data. Phylogenetic relationships among HVSI and genomic mtDNA sequences were established using the reduced median network algorithm [70]. Ages of clades were estimated using the rho statistic [71], and a calibration of 1 transition within np 16090–16365 corresponds to 20,180 years [72] for HVSI sequences.

For population comparisons, F_ST _distances were calculated based on haplogroup and haplotype frequencies using Arlequin 2.0 [73]. In order to diminish the strong influence of the common haplotypes in F_ST _distances, an additional measure of haplotypic identity [I_HT _= (HT_XY_/(HT_X_·HT_Y_)] was used, where HT_XY _is the number of shared haplotypes between populations X and Y, and HT_X _and HT_Y _are the numbers of different haplotypes in the populations X and Y, respectively. Multidimensional scaling (MDS) plots were obtained from F_ST _distances and principal component analysis (PCA) from haplogroup frequencies using SPSS version 13.0 (SPSS Inc., Chicago, Illinois).

## Accession numbers

The six new complete mitochondrial DNA sequences are registered under GenBank accession numbers: FJ236978–FJ236983.

## Authors' contributions

HE, VMC, AMG, ABEG and JML conceived the project, designed the experiments and wrote the paper; HE, MB, RB, ND, JML collected the samples; HE, MB, RB, ND and VMC extracted the DNA; HE, VMC, AMG, KKAA and JML performed the experiments, sequencing aligning and haplogroups typing; HE, VMC, AMG and JML analyzed the data and performed the statistical analysis. All authors read, revised and approved the final manuscript.

## Supplementary Material

Additional file 1 **H haplotypes detected in the different populations analyzed. **The data show the H-subgroup assignation, and HVS I and HVS II sequences of individuals analyzed in the following studied populations: Galicia, Castilla-Mancha, Castilla-León, Andalusia, Santander, León, Potes, Pasiegos, Maragatos, Tunisian Berbers, Tunisia, Libya, Algeria, Algeria Berbers, Moroccan Berbers, Morocco, Sahara, Mauritania, Saudi-Arabia, Jordan, Dead Sea, Palestine, Syria, Iran. Click here for file

Additional file 2 **Origin, localization, sample size, number of H, number of H classified in subhaplogroups, and percentage of H in different populations.** The table list the populations and geographic areas used to search for H haplotypes assigned to an H subgroup. Origin, localization, sample size, number of H, number of H classified in subhaplogroups, percentage of H and references are shown. Click here for file

Additional file 3 **Diagnostic mt SNPs, RFLPs and primers used to detect the different H subhaplogroups.** Table shows the diagnostic mt SNPs, RFLPs and primers used to detect the different H subhaplogroups in all or some of the samples. Click here for file

## References

[B1] N. Newman JL (1995). The peopling of Africa A geographic interpretation.

[B2] Garcea EA, Giraudi C (2006). Late Quaternary human settlement patterning in the Jebel Gharbi. J Hum Evol.

[B3] Straus LG (2001). Africa and Iberia in the Pleistocene. Quaternary International.

[B4] Bosch E, Calafell F, Pérez-Lezaun A, Clarimón J, Comas D, Mateu E, Martínez-Arias R, Morera B, Brakez Z, Akhayat O, Sefiani A, Hariti G, Cambon-Thomsen A, Bertranpetit J (2000). Genetic structure of north-west Africa revealed by STR analysis. Eur J Hum Genet.

[B5] Flores C, Hernández M, González AM, Cabrera VM, Arnaiz-Villena A (2000). Genetic affinities among human populations inhabiting the Subsaharan area, Northwest Africa, and the Iberian Peninsula. Procedings of Prehistoric Iberia: Genetics, Anthropology, and Linguistics.

[B6] Bosch E, Calafell F, Pérez-Lezaun A, Comas D, Mateu E, Bertranpetit J (1997). Population history of north Africa: evidence from classical genetic markers. Hum Biol.

[B7] Barbujani G, Pilastro A, De Domenico S, Renfrew C (1994). Genetic variation in North Africa and Eurasia: Neolithic demic diffusion vs. Paleolithic Colonisation. Am J Phys Anthropol.

[B8] Richards M, Macaulay V, Hickey E, Vega E, Sykes B, Guida V, Rengo C, Sellitto D, Cruciani F, Kivisild T, Villems R, Thomas M, Rychkov S, Rychkov O, Rychkov Y, Golge M, Dimitrov D, Hill E, Bradley D, Romano V, Cali F, Vona G, Demaine A, Papiha S, Triantaphyllidis C, Stefanescu G, Hatina J, Belledi M, Di Rienzo A, Novelletto A, Oppenheim A, Norby S, Al-Zaheri N, Santachiara-Benerecetti S, Scozari R, Torroni A, Bandelt HJ (2000). Tracing European founder lineages in the Near Eastern mtDNA pool. Am J Hum Genet.

[B9] Maca-Meyer N, González AM, Larruga JM, Flores C, Cabrera VM (2001). Major genomic mitochondrial lineages delineate early human expansions. BMC Genetics.

[B10] Herrnstadt C, Elson JE, Fahy E, Preston G, Turnbull DM, Anderson C, Ghosh SS, Olefsky JM, Beal MF, Davis RE, Howell N (2002). Reduced median-network analysis of complete mitochondrial DNA coding-region sequences for the major African, Asian and European haplogroups. Am J Hum Genet.

[B11] Maca-Meyer N, González AM, Pestano J, Flores C, Larruga JM, Cabrera VM (2003). Mitochondrial DNA transit between West Asia and North Africa inferred from U6 phylogeography. BMC Genetics.

[B12] Reidla M, Kivisild T, Metspalu E, Kaldma K, Tambets K, Tolk HV, Parik J, Loogväli EL, Derenko M, Malyarchuk B, Bermisheva M, Zhadanov S, Pennarun E, Gubina M, Golubenko M, Damba L, Fedorova S, Gusar V, Grechanina E, Mikerezi I, Moisan JP, Chaventré A, Khusnutdinova E, Osipova L, Stepanov V, Voevoda M, Achilli A, Rengo C, Rickards O, De Stefano GF, Papiha S, Beckman L, Janicijevic B, Rudan P, Anagnou N, Michalodimitrakis E, Koziel S, Usanga E, Geberhiwot T, Herrnstadt C, Howell N, Torroni A, Villems R (2003). Origin and diffusion of mtDNA haplogroup X. Am J Hum Genet.

[B13] Olivieri A, Achilli A, Pala M, Battaglia V, Fornarino S, Al-Zahery N, Scozzari R, Cruciani F, Behar DM, Dugoujon JM, Coudray C, Santachiara-Benerecetti AS, Semino O, Bandelt HJ, Torroni A (2006). The mtDNA legacy of the Levantine early Upper Palaeolithic in Africa. Science.

[B14] González AM, Larruga JM, Abu-Amero KK, Shi Y, Pestano J, Cabrera VM (2007). Mitochondrial lineage M1 traces an early human backflow to Africa. BMC Genomics.

[B15] Rando JC, Pinto F, González AM, Hernández M, Larruga JM, Cabrera VM, Bandelt HJ (1998). Mitochondrial DNA analysis of Northwest African populations reveals genetic exchanges with European, Near-Eastern, and sub-Saharan populations. Ann Hum Genet.

[B16] Krings M, Halim Salem A, Bauer K, Geisert H, Malek AK, Chaix L, Simon C, Welsby D, Di Rienzo A, Utermann G, Sajantila A, Pääbo S, Stoneking M (1999). mtDNA analysis of Nile valley populations: A genetic corridor or a barrier to migration?. Am J Hum Genet.

[B17] Macaulay VA, Richards MB, Hickey L, Vega E, Cruciani F, Guida V, Scozzari R, Bonné-Tamir B, Sykes B, Torroni A (1999). The emerging tree of West Eurasian mtDNAs: a synthesis of control-region sequences and RFLPs. Am J Hum Genet.

[B18] Brakez Z, Bosch E, Izaabel H, Akhayat O, Comas D, Bertranpetit J, Calafell F (2001). Human mitochondrial DNA sequence variation in the Moroccan population of the Souss area. Hum Biol.

[B19] Plaza S, Calafell F, Helal A, Bouzerna N, Lefranc G, Bertranpetit J, Comas D (2003). Joining the pillars of Hercules: mtDNA sequences show multidirectional gene flow in the western Mediterranean. Ann Hum Genet.

[B20] Fadhlaoui-Zid K, Plaza S, Calafell F, Ben Amor M, Comas D, Bennamar El Gaaied A (2004). Mitochondrial DNA heterogeneity in Tunisian Berbers. Ann Hum Genet.

[B21] Stevanovitch A, Gilles A, Bouzaid E, Kefi R, Paris F, Gayraud RP, Spadoni JL, El-Chenawi F, Béraud-Colomb E (2004). Mitochondrial DNA sequence diversity in a sedentary population from Egypt. Ann Hum Genet.

[B22] Cherni L, Loueslati BY, Pereira L, Ennafaa H, Amorim A, El Gaaied AB (2005). Female gene pools of Berber and Arab neighboring communities in central Tunisia: microstructure of mtDNA variation in North Africa. Hum Biol.

[B23] Loueslati BY, Cherni L, Khodjet-Elkhil H, Ennafaa H, Pereira L, Amorim A, Ben Ayed F, Ben Ammar Elgaaied A (2006). Islands inside an island: reproductive isolates on Jerba island. Am J Hum Biol.

[B24] Torroni A, Bandelt HJ, D'Urbano L, Lahermo P, Moral P, Sellitto D, Rengo C, Forster P, Savontaus ML, Bonné-Tamir B, Scozzari R (1998). mtDNA analysis reveals a major late Paleolithic population expansion from southwestern to northeastern Europe. Am J Hum Genet.

[B25] Torroni A, Bandelt HJ, Macaulay V, Richards M, Cruciani F, Rengo C, Martinez-Cabrera V, Villems R, Kivisild T, Metspalu E, Parik J, Tolk HV, Tambets K, Forster P, Karger B, Francalacci P, Rudan P, Janicijevic B, Rickards O, Savontaus ML, Huoponen K, Laitinen V, Koivumäki S, Sykes B, Hickey E, Novelletto A, Moral P, Sellitto D, Coppa A, Al-Zaheri N, Santachiara-Benerecetti AS, Semino O, Scozzari R (2001). A signal, from human mtDNA, of postglacial recolonization in Europe. Am J Hum Genet.

[B26] Achilli A, Rengo C, Battaglia V, Pala M, Olivieri A, Fornarino S, Magri C, Scozzari R, Babudri N, Santachiara-Benerecetti AS, Bandelt HJ, Semino O, Torroni A (2005). Saami and Berbers-an unexpected mitochondrial DNA link. Am J Hum Genet.

[B27] Kivisild T, Reidla M, Metspalu E, Rosa A, Brehm A, Pennarun E, Parik J, Geberhiwot T, Usanga E, Villems R (2004). Ethiopian mitochondrial DNA heritage: tracking gene flow across and around the gate of tears. Am J Hum Genet.

[B28] Abu-Amero KK, González AM, Larruga JM, Bosley TM, Cabrera VM (2007). Eurasian and African mitochondrial DNA influences in the Saudi Arabian population. BMC Evol Biol.

[B29] Abu-Amero KK, Larruga JM, Cabrera VM, González AM (2008). Mitochondrial DNA structure in the Arabian Peninsula. BMC Evol Biol.

[B30] Bosch E, Calafell F, Comas D, Oefner PJ, Underhill PA, Bertranpetit J (2001). High-resolution analysis of human Y-chromosome variation shows a sharp discontinuity and limited gene flow between northwestern Africa and the Iberian Peninsula. Am J Hum Genet.

[B31] Arredi B, Poloni ES, Paracchini S, Zerjal T, Fathallah DM, Makrelou M, Pascali VL, Novelletto A, Tyler-Smith C (2004). A predominantly neolithic origin for Y-chromosomal DNA variation in North Africa. Am J Hum Genet.

[B32] Cruciani F, La Fratta R, Santolamazza P, Sellitto D, Pascone R, Moral P, Watson E, Guida V, Colomb EB, Zaharova B, Lavinha J, Vona G, Aman R, Cali F, Akar N, Richards M, Torroni A, Novelletto A, Scozzari R (2004). Phylogeographic analysis of haplogroup E3b (E-M215) Y chromosomes reveals multiple migratory events within and out of Africa. Am J Hum Genet.

[B33] Semino O, Magri C, Benuzzi G, Lin AA, Al-Zahery N, Battaglia V, Maccioni L, Triantaphyllidis C, Shen P, Oefner PJ, Zhivotovsky LA, King R, Torroni A, Cavalli-Sforza LL, Underhill PA, Santachiara-Benerecetti AS (2004). Origin, diffusion, and differentiation of Y-chromosome haplogroups E and J: inferences on the neolithization of Europe and later migratory events in the Mediterranean area. Am J Hum Genet.

[B34] Cruciani F, La Fratta R, Trombetta B, Santolamazza P, Sellitto D, Colomb EB, Dugoujon JM, Crivellaro F, Benincasa T, Pascone R, Moral P, Watson E, Melegh B, Barbujani G, Fuselli S, Vona G, Zagradisnik B, Assum G, Brdicka R, Kozlov AI, Efremov GD, Coppa A, Novelletto A, Scozzari R (2007). Tracing past human male movements in northern/eastern Africa and western Eurasia: new clues from Y-chromosomal haplogroups E-M78 and J-M12. Mol Biol Evol.

[B35] Wood ET, Stover DA, Ehret C, Destro-Bisol G, Spedini G, McLeod H, Louie L, Bamshad M, Strassmann BI, Soodyall H, Hammer MF (2005). Contrasting patterns of Y chromosome and mtDNA variation in Africa: evidence for sex-biased demographic processes. Eur J Hum Genet.

[B36] Finnila S, Lehtonen MS, Majamaa K (2001). Phylogenetic network for European mtDNA. Am J Hum Genet.

[B37] Achilli A, Rengo C, Magri C, Battaglia V, Olivieri A, Scozzari R, Cruciani F, Zeviani M, Briem E, Carelli V, Moral P, Dugoujon JM, Roostalu U, Loogväli EL, Kivisild T, Bandelt HJ, Richards M, Villems R, Santachiara-Benerecetti AS, Semino O, Torroni A (2004). The molecular dissection of mtDNA haplogroup H confirms that the Franco-Cantabrian glacial refuge was a major source for the European gene pool. Am J Hum Genet.

[B38] Loogväli EL, Roostalu U, Malyarchuk BA, Derenko MV, Kivisild T, Metspalu E, Tambets K, Reidla M, Tolk HV, Parik J, Pennarun E, Laos S, Lunkina A, Golubenko M, Barac L, Pericic M, Balanovsky OP, Gusar V, Khusnutdinova EK, Stepanov V, Puzyrev V, Rudan P, Balanovska EV, Grechanina E, Richard C, Moisan JP, Chaventré A, Anagnou NP, Pappa KI, Michalodimitrakis EN, Claustres M, Gölge M, Mikerezi I, Usanga E, Villems R (2004). Disuniting uniformity: a pied cladistic canvas of mtDNA haplogroup H in Eurasia. Mol Biol Evol.

[B39] Quintáns B, Alvarez-Iglesias V, Salas A, Phillips C, Lareu MV, Carracedo A (2004). Typing of mitochondrial DNA coding region SNPs of forensic and anthropological interest using SNaPshot minisequencing. Forensic Sci Int.

[B40] Pereira L, Richards M, Gozos A, Alonso A, Albarrán C, Garcia O, Behar DM, Gölge M, Hatina J, Al-Gazali L, Bradley DG, Macaulay V, Amorim A (2005). High-resolution mtDNA evidence for the late-glacial resettlementof Europe from an Iberian refugium. Genome Res.

[B41] Corte-Real HB, Macaulay VA, Richards MB, Hariti G, Issad MS, Cambon-Thomsen A, Papiha S, Bertranpetit J, Sykes BC (1996). Genetic diversity in the Iberian Peninsula determined from mitochondrial sequence analysis. Ann Hum Genet.

[B42] González AM, Cabrera VM, Larruga JM, Tounkara A, Noumsi G, Thomas BN, Moulds JM (2006). Mitochondrial DNA Variation in Mauritania and Mali and their Genetic Relationship to Other Western Africa Populations. Ann Hum Genet.

[B43] Lutz S, Weisser HJ, Heizmann J, Pollak S (1998). Location and frequency of polymorphic positions in the mtDNA control region of individuals from Germany. Int J Legal Med.

[B44] Larruga JM, Diez F, Pinto FM, Flores C, González AM (2001). Mitochondrial DNA characterisation of European isolates: the Maragatos from Spain. Eur J Hum Genet.

[B45] Pult I, Sajantila A, Simanainem J, Georgiev O, Schaffner W, Paabo S (1994). Mitochondrial DNA sequences from Switzerland Reveal striking Homogeneity of European Populations. Biol Chem Hoppe Seyler.

[B46] Tagliabracci A, Turchi C, Buscemi L, Sassaroli C (2001). Polymorphism of the mitochondrial DNA control region in Italians. Int J Legal Med.

[B47] Zupanic Pajnic I, Balazic J, Komel R (2004). Sequence polymorphism of the mtDNA control region in the Slovenian population. Int J Legal Med.

[B48] Richard C, Pennarun E, Kivisild T, Tambets K, Tolk HV, Metspalu E, Reidla M, Chevalier S, Giraudet S, Lauc LB, Pericic M, Rudan P, Claustres M, Journel H, Dorval I, Müller C, Villems R, Chaventré A, Moisan JP (2007). An mtDNA perspective of French genetic variation. Ann Hum Biol.

[B49] Brandstaetter A, Zimmermann B, Wagner J, Goebel T, Roeck AW, Salas A, Carracedo A, Parson W (2008). Timing and deciphering mitochondrial DNA macro-haplogroup R0 variability in Central Europe and Middle East. BMC Evol Biol.

[B50] Voevoda MI, Avksentyuk AV, Ivanova AV, Astakhova TI, Babenko VN, Kurilovich SA, Duffy LK, Segal B, Shields GF (1994). Molecular genetic studies in the population of native inhabitants of Chukchee Peninsula. Analysis of polymorphism of mitochondrial DNA and of genes controlling alcohol metabolizing enzymes. Sibirskii Ekolog Z.

[B51] Kolman CJ, Sambuughin N, Bermingham E (1996). Mitochondrial DNA analysis of Mongolian populations and implications for the origin of New World founders. Genetics.

[B52] Comas D, Calafell F, Mateu E, Perez-Lezaun A, Bosch E, Martinez-Arias R, Clarimon J, Facchini F, Fiori G, Luiselli D, Pettener D, Bertranpetit J (1998). Trading genes along the silk road: mtDNA sequences and the origin of central Asian populations. Am J Hum Genet.

[B53] Al-Zahery N, Semino O, Benuzzi G, Magri C, Passarino G, Torroni A, Santachiara-Benerecetti AS (2003). Y-chromosome and mtDNA polymorphisms in Iraq, a crossroad of the early human dispersal and of post-Neolithic migrations. Mol Phylogenet Evol.

[B54] Metspalu M, Kvisild T, Metspalu E, Parik J, Hudjashov G, Kaldma K, Serk P, Karmin M, Behar DM, Thomas M, Gilbert P, Endicott P, Mastana S, Papiha SS, Skorecki K, Torroni A, Villems R (2004). Most of the extant mtDNA boundaries in South and Southwest Asia were likely shaped during the initial settlement of Eurasia by anatomically modern humans. BMC Genetics.

[B55] Tomas C, Sánchez JJ, Barbaro A, Brandt-Casadevall C, Hernández A, Ben Diva M, Ramón M, Morling N (2008). X-chromosome SNP analyses in 11 human Mediterranean populations show a high overall genetic homogeneity except in North-west Africans (Moroccans). BMC Evol Biol.

[B56] Comas D, Calafell F, Benchemsi N, Helal A, Lefranc G, Stoneking M, Batzer MA, Bertranpetit J, Sajantila A (2000). Alu insertion polymorphisms in MW Africa and the Iberian Peninsula: evidence for a strong genetic boundary through the Gibraltar Straits. Hum Genet.

[B57] Ennafaa H, Amor MB, Yacoubi-Loueslati B, Khodjet el-khil H, Gonzalez-Perez E, Moral P, Maca-Meyer N, Elgaaied A (2006). Alu polymorphisms in Jerba Island population (Tunisia): comparative study in Arab and Berber groups. Ann Hum Biol.

[B58] Flores C, Maca-Meyer N, Pérez JA, Hernández M, Cabrera VM (2001). Y-chromosome differentiation in Northwest Africa. Hum Biol.

[B59] Khodjet el Khil H, Marrakchi RT, Loueslati BY, Langaney A, Fellous M, BenAmmar Elgaaied A (2005). Distribution of Y chromosome lineages in Jerba island population. Forensic Sci Int.

[B60] Flores C, Maca-Meyer N, González AM, Cabrera VM (2000). Northwest African distribution of the CD4/Alu microsatellite haplotypes. Ann Hum Genet.

[B61] Esteban E, González-Pérez E, Harich N, López-Alomar A, Via M, Luna F, Moral P (2004). Genetic relationships among Berbers and South Spaniards based on CD4 microsatellite/Alu haplotypes. Ann Hum Biol.

[B62] Mishmar D, Ruiz-Pesini E, Golik P, Macaulay V, Clark AG, Hosseini S, Brandon M, Easley K, Chen E, Brown MD, Sukernik RI, Olckers A, Wallace DC (2003). Natural selection shaped regional mtDNA variation in humans. P Natl Acad Sci USA.

[B63] Nebel A, Landau-Tasseron E, Filon D, Oppenheim A, Faerman M (2002). Genetic evidence for the expansion of Arabian tribes into the Southern Levant and North Africa. Am J Hum Genet.

[B64] Anderson S, Bankier AT, Barrell BG, de Bruijn MH, Coulson AR, Drouin J, Eperon IC, Nierlich DP, Roe BA, Sanger F, Schreier PH, Smith AJ, Staden R, Young IGF (1981). Sequence and organization of the human mitochondrial genome. Nature.

[B65] Andrews RM, Kubacka I, Chinnery PF, Lightowlers RN, Turnbull DM, Howell N (1999). Reanalysis and revision of the Cambridge reference sequence for human mitochondrial DNA. Nat Genet.

[B66] Howell N, Oostra RJ, Bolhuis PA, Spruijt L, Clarke LA, Mackey DA, Preston G, Herrnstadt C (2003). Sequence analysis of the mitochondrial genomes from Dutch pedigrees with Leber hereditary optic neuropathy. Am J Hum Genet.

[B67] Fraumene C, Belle EM, Castri L, Sanna S, Mancosu G, Cosso M, Marras F, Barbujani G, Pirastu M, Angius A (2006). High Resolution Analysis and Phylogenetic Network Construction Using Complete mtDNA Sequences in Sardinian Genetic Isolates. Mol Biol Evol.

[B68] Roostalu U, Kutuev I, Loogväli EL, Metspalu E, Tambets K, Reidla M, Khusnutdinova EK, Usanga E, Kivisild T, Villems R (2007). Origin and expansion of haplogroup H, the dominant human mitochondrial DNA lineage in West Eurasia: the Near Eastern and Caucasian perspective. Mol Biol Evol.

[B69] Nei M (1987). Molecular evolutionary genetics.

[B70] Bandelt HJ, Forster P, Röhl A (1999). Median-joining networks for inferring intraspecific phylogenies. Mol Biol Evol.

[B71] Saillard J, Forster P, Lynnerup N, Bandelt HJ, Norby S (2000). mtDNA variation among Greenland eskimos: the edge of the Beringian expansion. Am J Hum Genet.

[B72] Forster P, Harding R, Torroni A, Bandelt HJ (1996). Origin and evolution of Native American mtDNA variation: a reappraisal. Am J Hum Genet.

[B73] Schneider S, Roessli D, Excoffier L Arlequin ver. 2.000: A software for population genetics data analysis. Genetics and Biometry Laboratory.

